# A Virtual Testing Framework for Real-Time Validation of Automotive Software Systems Based on Hardware in the Loop and Fault Injection

**DOI:** 10.3390/s24123733

**Published:** 2024-06-08

**Authors:** Mohammad Abboush, Christoph Knieke, Andreas Rausch

**Affiliations:** Institute for Software and Systems Engineering, Technical University of Clausthal, 38678 Clausthal-Zellerfeld, Germany; christoph.knieke@tu-clausthal.de (C.K.); andreas.rausch@tu-clausthal.de (A.R.)

**Keywords:** automotive software systems, virtual test, real-time systems, safety validation, fault injection, hardware in the loop (HIL), system integration and test, model-based development

## Abstract

To validate safety-related automotive software systems, experimental tests are conducted at different stages of the V-model, which are referred as “X-in-the-loop (XIL) methods”. However, these methods have significant drawbacks in terms of cost, time, effort and effectiveness. In this study, based on hardware-in-the-loop (HIL) simulation and real-time fault injection (FI), a novel testing framework has been developed to validate system performance under critical abnormal situations during the development process. The developed framework provides an approach for the real-time analysis of system behavior under single and simultaneous sensor/actuator-related faults during virtual test drives without modeling effort for fault mode simulations. Unlike traditional methods, the faults are injected programmatically and the system architecture is ensured without modification to meet the real-time constraints. Moreover, a virtual environment is modeled with various environmental conditions, such as weather, traffic and roads. The validation results demonstrate the effectiveness of the proposed framework in a variety of driving scenarios. The evaluation results demonstrate that the system behavior via HIL simulation has a high accuracy compared to the non-real-time simulation method with an average relative error of 2.52. The comparative study with the state-of-the-art methods indicates that the proposed approach exhibits superior accuracy and capability. This, in turn, provides a safe, reliable and realistic environment for the real-time validation of complex automotive systems at a low cost, with minimal time and effort.

## 1. Introduction

Today’s software-driven automotive systems are considered heterogeneous and complex, and have a high degree of functional interdependencies [1]. The emergence of intelligent assistance functions, such as Advanced Driver Assistance Systems (ADASs) [2], has increased the complexity of the system architecture. In a modern vehicle, the system architecture includes up to 120 ECUs communicating over more than five system buses and exchanging 2 million messages per minute [3,4]. Testing such complex systems against functional and non-functional requirements is therefore a challenge in the industry. In addition, automotive safety systems in particular must be rigorously verified and validated according to the functional safety standard ISO 26262 [5]. In the development cycle of ISO 26262, different test levels are defined, i.e., unit test, integration test and system test [6]. It also provides requirements and recommendations for the development process to ensure the functional safety of the developed systems.

At the vehicle integration and testing level, open-road tests, i.e., real-world test drives, are conducted to detect unexpected faults that have not been detected in the design phases. In such a test approach, a real vehicle prototype is used by manufacturers to validate the integration of vehicle subsystems, including real ECUs, sensors and actuators, and their networks, on public roads [7]. Meanwhile, data acquisition systems capture system behavior as multivariate time series data under realistic conditions. Despite the advantages of the real test drive in terms of high test coverage [8], it suffers from several burdens:

1. To meet the requirements of ISO 26262, it is difficult to perform a large number of representative and relevant test kilometers [9].
2. The higher the coverage of critical test situations, the higher the probability of a risk to the test driver during the tests [10].
3. Validation of the system under fault conditions using fault injection methods is not feasible [11].
4. The ability to reproduce an accurate test experiment is a challenge due to the high cost per test mile, time and uncontrolled environment [12].
5. Finally, manual analysis of the huge amount of data recorded during road tests based on expert knowledge is costly, time consuming and labor intensive [13,14].

Therefore, a safe, reliable and flexible test framework is required to accelerate the test process, reduce the time and effort, and cover the critical scenarios. Recently, virtual methods have contributed to improve the V-cycle development in the automotive industry in terms of verification and validation [15]. To overcome the limitations of real test drives, various virtual testing and validation methods, known as “X-in-the-loop” testing, have been introduced [16]. Among them, model-in-the-loop (MIL) [17], software-in-the-loop (SIL) [18], processor-in-the-loop (PIL) [19], hardware-in-the-loop (HIL) [20] and vehicle-in-the-loop (VIL) [21] have emerged in recent years. Depending on the virtual environment, the controller in the aforementioned methods is connected to the controlled system in a closed loop [22].

In MIL, the behavioral models of the controller and the plant are developed together at the same level according to the specifications using a simulation environment, e.g., MATLAB/ Simulink [23]. In this way, verification and validation of the System Under Test (SUT) can be performed efficiently at an early stage of the development process. To validate the system robustness and reduce potential risks, system behavior under abnormal conditions is analyzed using model-based fault injection (FI) [24]. Notably, according to the functional safety standard ISO 26262 [5], abnormal conditions that can lead to the failure of an element or object are called faults. Therefore, in our study, the term abnormal conditions was used to refer to the system behavior in the presence of faults in the system components, i.e., sensors and actuators. Normal condition, on the other hand, refers to the system behavior under fault-free conditions (“golden run”).

Once the functional logic architecture test is complete, SIL is performed to validate the generated control model code against the requirements, taking into account its interaction with the controlled system model. The goal of this test method is to verify the correctness of the production code in a virtual environment, focusing on the specifications of each component. This allows for the unlimited validation of multiple versions of control algorithms in the system environment and the ability to perform a large number of test runs. By reducing the cost and increasing the speed of the test process, MIL and SIL are superior to other methods in terms of early validation of the developed control strategy without requiring real hardware. However, since the control model/code and the plant are executed at the same simulation level, i.e., on the host PC, the real-time constraints and physical communication modeling are not considered. In addition, the conditions of the real environment and the target machine differ from those of the simulation environment, which in turn leads to deviations in test accuracy.

On the right side of the V-model, PIL is used to validate the executable object code in the processor. The compiled control code is deployed and executed on the embedded processor connected in the loop to the plant model. Despite the benefits of real-time control validation, considering real-time constraints, controlled system execution is limited to the non-real-time method, i.e., on a host PC.

The VIL method is used in a later phase of the mode-based development approach, where a real vehicle prototype is used to achieve a high degree of realism in vehicle behavior. In general, the VIL method is used to validate the target system against functional safety requirements (FSRs), taking into account driver behavior [25]. To avoid the risk of a real test drive on public roads, a virtual simulation environment [26] is used in which dynamic traffic with pedestrians can be modeled and displayed in a 3D visualization. However, the more complex the preparation of the VIL method, the higher the cost and time required, especially for complex systems.

Recently, HIL simulation has been introduced as the best solution to the aforementioned method issues, i.e., the time constraints and the complexity of the real vehicle prototype. HIL is characterized by high reliability and flexibility of the test environment, as well as low cost and time. The ability to execute the generated code of the control and plant model in real time allows comprehensive system behavior to be accurately captured and simulated. Moreover, critical scenarios and failures can be investigated without compromising the safety of the test engineer. In addition to the reproduced and automated test process, real physical components, e.g., sensors, actuators and steering system, can be connected to HIL to enable reliable validation at an early stage before production. In addition, FI based on HIL simulation paved the way for efficient system-level validation of functional safety in critical edge cases according to the ISO 26262 standard [27].

The current state-of-the-art test methods do not ensure real-time conditions with HIL simulation. The reason is that the FI method is implemented by extending the system architecture with an additional simulation block to model the fault mode. However, the execution tasks of the added block require additional time, which in turn leads to a violation of real-time constraints during system execution on HIL. Moreover, in the case of complex systems, achieving a high coverage of fault modeling in different system components is not feasible. The shortcomings of the current simulation methods can be listed as follows:

- Ignoring real-time requirements and being unable to replicate the interaction of physical hardware in the case of a pure non-real-time simulation, i.e., MIL and SIL.
- Inability to efficiently troubleshoot network-related issues as in the case of PIL without connection to real hardware components.
- The high modeling effort with the potential occurrence of real-time violations when the system is validated under fault conditions using HIL simulation.
- Safety risks, high costs and the complexity of integrating real vehicle components in VIL tests.

To overcome the aforementioned drawbacks, a novel real-time test framework based on HIL simulation and fault injection is proposed in this study. Unlike the traditional validation methods, the proposed approach allows FI to be performed programmatically in real time via the CAN bus interface, keeping the structure of the system as a black box unchanged. In addition to the high coverage of possible fault types and locations, the analysis of the system behavior in case of faults during the virtual test drive by the user is enabled. The key objective of the framework is to validate the SUT under normal and abnormal conditions of the system components considering the driver behavior. In addition, a dynamic traffic environment is modeled and visualized as a 3D visualization to achieve a high degree of realism. To demonstrate the advantages and capabilities of the proposed framework, a high-fidelity entire vehicle model, including a complex gasoline engine model with vehicle dynamics and drivetrain, is used as a case study. It is essential to mention that the proposed framework is also applicable to other vehicle systems/subsystems, e.g., from internal combustion engines to electric or hybrid vehicles, as the development and validation process is unique. Since the FI process takes place at the CAN bus interface, only the configurations of the FI attributes should be specified to analyze the fault effects on other systems. To the best of our knowledge, this is the first time that such a problem has been investigated. Furthermore, no specific studies have been conducted to date that address this issue considering different road and environmental conditions during virtual test drives.

The contributions of this study can be summarized as follows:

- Design and implementation of a virtual test framework for performing real-time validation activities at the system integration and testing phase of the V-model development process.
- Development of a novel driving simulator capable of analyzing the system behavior in real time for safety validation purposes in case of fault occurrence, i.e., sensor/ actuator-related faults.
- High degree of coverage of the system components as the location of fault injection, including several subsystems in the vehicle system.
- Since the HIL operates as a black-box test, different types of faults can be injected programmatically and automatically, individually or simultaneously, without changing the original system model.
- Development of a driving environment with 3D visualization, taking into account the effect of dynamic traffic, dynamic objects and weather conditions on system behavior.

The body of the article is organized as follows. Section 2 provides an overview of related work, highlighting the main contributions of the proposed work in comparison to other research. Section 3 contains a description of the proposed method with emphasis on the main structural phases. Section 4 presents the hardware and software architecture of the proposed framework, including the development phases. The experimental and evaluation results are discussed in Section 5. Finally, Section 6 presents the conclusions and future work.

## 2. Related Work

Recently, virtual testing has attracted the attention of researchers in various fields to overcome the shortcomings of real test drives in terms of safety, cost and effort [28,29]. The scope of this approach is not limited to the validation of the developed control strategy [10,30], but also extends to the development of human–machine interfaces [31]. In addition, quality assurance aspects of the developed overall system, e.g., ADAS, can be effectively ensured while maintaining low cost and complexity [32,33].

Various methods have been employed to implement the virtual driving system, based on pure simulation tools, real prototypes or real-time simulation systems. For example, Saraoğlu et al. [34] have investigated the flexibility of the model-based development approach to propose a simulation framework for analyzing the safety characteristics of autonomous vehicle functions. In this work, considering the traffic model and driving scenarios, the FI method was used to validate the functional safety requirements of the target system. Furthermore, the fault propagation between the system components has been analyzed in the Simulink environment. Despite the demonstrated applicability of the proposed framework for systematic safety evaluation, the method is limited to non-real-time executions. Furthermore, manual driving is not considered in the simulation framework, which does not capture the driver’s behavior. In the same context, a validation methodology for decision and control strategies has been developed in [35] with a focus on automated driving ECUs. Based on the Dynacar simulation tool, the results show a high degree of modularity and adaptability not only in the controller selection, but also in the scenario and vehicle type configurations. A 3D visualization of the driving environment was also modeled, taking into account the route with different roundabouts and intersections. Similarly, to enable the validation process in real time, Sievers et al. [36] proposed a unified toolchain for autonomous vehicle testing. The advantages of the proposed tools were demonstrated in the validation of sensor-based ECUs at different test levels, i.e., SIL and HIL. Camera raw data injection has been used as an example setup to demonstrate the applicability of the proposed tool. However, the proposed work is limited to the validation process under normal behavior. In our study, on the other hand, the safety and reliability of the SUT has been evaluated in the case of fault occurrence considering hazardous situations.

To overcome the limitations of simulation-based non-real-time testing in evaluating the SUT under real operating conditions, various research works have been conducted to improve the VIL simulator. By doing so, the integration of real components with the SUT can be effectively validated considering the real driving and virtual environment for various automotive applications [37]. As an example, Park et al. proposed a modular VIL topology for the validation of ADAS applications in [38]. In addition to the 3D virtual environment and sensor emulation, a real vehicle was used to test the real-time stability of the ADAS. The results highlight the advantages of the proposed topology in terms of maintenance time and cost savings, as well as the high degree of driving realism. In this context, in [39], a novel adaptive cruise control (ACC) system with a fuzzy-based controller has been developed and validated considering various weather conditions and road tire friction. The core concept of the proposed study is to generate a dynamic reference signal so that the safe relative distance and speed of the vehicle can be adjusted, resulting in high safety and comfort performance. Although the validation results in various driving maneuvers show the effectiveness of the proposed approach, the validation of the target system in the presence of faults in the system components has not been explored. To exploit the advantages of real road-based testing and pure simulation-based testing, Solmaz et al. in [40] proposed a testing methodology called Hybrid Testing for ADAS. Compared to the conventional ADAS testing approaches, the study presents the advantages of the proposed methodology in which the virtual environment with simulated vehicle components is combined with the real vehicle prototype. The architecture and structure of the co-simulation framework have been described and demonstrated using the use case of a lane change maneuver. Notably, in the aforementioned study, the ADAS functions, namely the trajectory planning algorithm, were considered as a single SUT and executed in the MicroAutoBox. However, the safety level for the operator in the case of failure is rather low in the mentioned studies. Moreover, the performance of the target system cannot be evaluated under faulty conditions of the components. In contrast, in our research, the reliability and safety characteristics of the SUT can be efficiently evaluated in real time, taking into account the abnormal environmental conditions and the failures of the system components.

As a result, there is a high demand for a test approach that can bridge the gap between real-world test drive, SIL and VIL in terms of cost, safety, reliability, repeatability and flexibility. To meet the above requirements, real-time HIL simulation has been proposed as the best solution. HIL simulation has achieved remarkable success in various applications [41]. Specifically, for the development of real-time driving simulators, several attempts have been proposed in the last decade [42,43,44]. For example, in [45], a signal HIL simulation platform focusing on electric vehicles was proposed to provide effective and accurate real-time validation of motion controllers. In this experimental platform, in addition to the possibility of manual driving, comprehensive characteristics of the target system were considered by modeling the vehicle dynamics in longitudinal, lateral and yaw directions. Furthermore, three driving scenarios were used to demonstrate the effectiveness of the proposed study, i.e., on a normal road, on a poor adhesion surface and on a slippery surface. However, unlike our study, the proposed platform does not take into account abnormal environmental conditions or failures due to faulty system components. Achieving the same goal, but for a different application, Chen et al. proposed a HIL simulation-based test platform in [46]. The focus of the proposed study is the validation of real ECUs for autonomous vehicle development. According to the proposed architecture, the platform consists of three layers, system modeling, multi-sensor simulation and virtual test environment layers. Similar to our work, the real ECU is connected to the simulated system via the CAN bus in a closed loop, considering the interaction of multiple agents and modeling scenarios as 3D visualization. However, in our work, in addition to the self-driving mode, manual driving was also enabled to consider the user’s behavior. Regarding the problem of validation of safety-related systems, i.e., the ABS and ESC systems, Tumasov et al. in [47] proposed a HIL test bench that enables virtual test driving. The proposed work is characterized by the connection of a real vehicle element, i.e., a hydraulic cylinder of the braking system, along with the real ECU and the dynamic model. The effectiveness of the proposed platform is validated by comparing the results of virtual and field tests of the same maneuver, taking into account the stability control requirements. However, significant damage to the system can occur if a failure occurs in the physical components. In contrast, in our proposed work, the physical components are simulated and implemented with a high fidelity simulation to accurately analyze the effects of faulty components in real time. Finally, the applicability of the HIL system to develop an integrated driving simulator (IDHIL) for the evaluation of cooperative eco-driving systems has been demonstrated in [48]. The HIL simulator, the network simulator and the driving simulator are the key components of the proposed work, in which ASM, MicroAutoBox and dSPACE tools were used. In addition, two use cases were used to demonstrate the effectiveness of the simulator, i.e., normal hybrid electric vehicles (HEVs) during simulation and connected hybrid electric vehicles (CHEVs) during eco-speed. However, the environment was limited to the predefined normal conditions of the dSPCAE tool, i.e., defined scenarios in ModelDesk, whereas in our work a generic environment was modeled, covering abnormal conditions and critical scenarios. Another difference lies in the applicability of our proposed framework to simulate component faults, e.g., sensors and actuators faults, to validate the response of the SUT in case of a failure. An overview of related work is given in Table 1.

In summary, the development of a generic validation framework that meets the requirements of flexibility, cost, coverage, repeatability and reliability has not been sufficiently explored. A review of existing work shows that most of the proposed work is limited to adapting test methods for specific goals during evaluation, without considering the fault conditions affecting SUTs. In addition, implementing the FI approach involves modifying the system architecture with additional blocks to model the faults that cause the real-time constraints to be violated.

Therefore, one of the main challenges facing the development of the virtual test approach is to simulate the system behavior under faults during the virtual real-time test drive while ensuring the structure of the SUT as a black box for real-time constraints. This challenge motivates us to investigate how to develop a real-time validation framework that meets these requirements and enables the safety and reliability analysis of the SUT under single and concurrent faults considering different environmental conditions. To this end, real-time HIL simulations, a high-fidelity full vehicle model, a virtual driving environment with high coverage of abnormal conditions, manual and self-driving, and real-time FI were used.

## 3. Methodology

This section presents the proposed methodology, highlighting the core hardware and software components and the communication between them.

In the automotive domain, the development process of safety-critical systems should comply with the relevant standard for functional safety, i.e., ISO 26262. In the proposed study, several aspects of testing in accordance with ISO 26262 have been taken into account. These include specifying test strategies, assessing the qualification of the test environment and verifying the suitability of the entire tool chain. The test process in the proposed approach follows a specified test strategy, that includes the definition of the test focus, the test object, the test level, the test method and the test environment. Since both HIL simulation and FI are explicitly recommended by ISO 26262 for the development process, they have been considered in the proposed approach. In addition, a software tool chain fully certified by TÜV SÜD, i.e., dSPACE tools, was used for the development process in the proposed approach.

**Figure 1:**
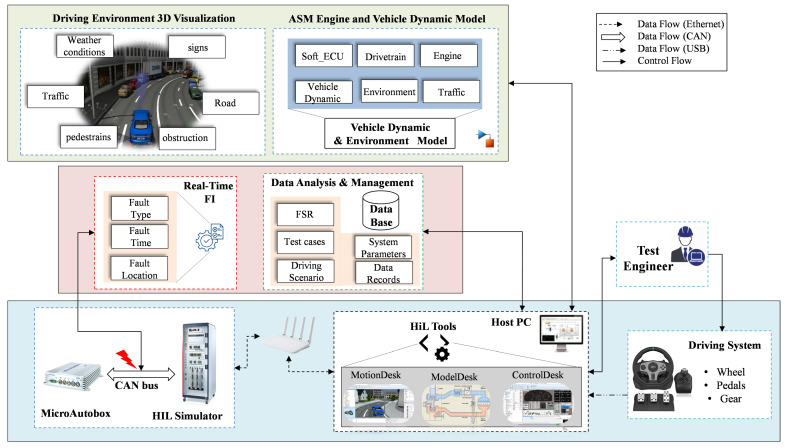
Proposed real-time validation framework based on HIL simulation and fault injection.

The architecture of the proposed framework consists of three different layers, namely the hardware component layer, the data analysis and FI layer, and the system model layer with environment visualization. Figure 1 illustrates the layers of the proposed framework architecture.

At the hardware component layer, the HIL simulation system is used to realize the simulation in real time, where the HIL simulator is directly connected to the real ECU. In this study, the control strategy is deployed and executed in the MicroAutoBox II, which acts as the real ECU, while the controlled system, representing the entire vehicle model, is executed in the HIL simulator. On the other hand, driving elements such as steering wheel, gearshift and pedals (accelerator, clutch and brake pedals) are employed to enable manual driving by the user. Remarkably, the driving system in the proposed approach supports both manual and automatic gears for manual driving. For real-time experiment setup and control, dSPCAE software tools are used on the host PC. MotionDesk, ModelDesk, ControlDesk and the FI GUI are used by the test engineer for parameterization, configuration, instrumentation, experiment execution, measurements and analysis. Thanks to the advantageous model-based design approach, the code is automatically generated from the models and implemented on the target machine via the host PC. The aforementioned hardware components of the proposed framework are interconnected via three communication protocols, namely Ethernet, CAN bus and USB. More specifically, the CAN bus is used to establish the interface signals between the controller and the controlled system, into which the disturbances are injected. On the other hand, the host PC is connected to the HIL system via Ethernet, which transfers the generated model code from the PC to the HIL elements. Finally, the driving commands of the HIL simulation controllers are transmitted to the host PC via the USB communication protocol.

To validate the robustness of the SUT and its performance under faulty conditions during execution, the real-time FI framework developed in previous work [49] is used in the second layer. For this purpose, three different attributes are identified, i.e., fault type, target location and injection time. According to the requirements and test objectives, the fault injector provides a list of sensor and actuator faults, i.e., gain, offset, stuck-at, delay, data loss, hard over, noise, drift and spike. Detailed information about the fault types can be found in [50]. Thanks to the CAN bus model feature, the signals from the SUT and the plant can be accessed during the real-time simulation. As a result, FI at the target component can be activated programmatically as a black-box test without changing the system model. Finally, the timing and duration of FI can be determined by the tester based on the standard system behavior, i.e., the desired drive cycle.

It is noteworthy that ISO 26262 does not define a specific location or type of faults to be injected into the target system. Instead, it mentions the potential system components that are prone to faults, including sensors, actuators and ECUs. Therefore, the selection of combinations of the faults’ attributes is derived from the FSR to be validated. Further details regarding the automated generation of fault test cases from FSR can be found in [51]. In the event that the FSR is affected by two faulty components, two locations are selected for the injection of the list of fault types. Specifically, two types of faults are injected into two different locations simultaneously and reciprocally in each experiment. Otherwise, a single location is selected for the injection of the list of fault types in each experiment.

Mathematically, the combination of faults is determined based on Equation (1). However, according to ISO 26262, the maximum affected points by fault are two locations. Consequently, any combination of more than two classes, e.g., three types at the same time, is disregarded.

(1) $$N_{c}=2^{d}-\left(d+1\right)$$

where $N_{c}$ represents the number of fault combinations and *d* is the number of fault types.

The mathematical representation of the sensor-related faults used in the study is presented in Equation (2) and Table 2.

(2) $$f^{\prime }\left(t\right)=g_{v}f\left(t\right)+s_{v}$$

where $f^{\prime }\left(t\right)$ is faulty or manipulated signal value and $g_{v}$ represents the gain value. The healthy or fault-free signal value is represented as f(t). $s_{v}$ represents the offset/bias value.

In addition to the FI framework, data analysis and management are also performed in the second layer. This contains the input and output of the target experiments. In other words, the functional and non-functional requirements are documented and analyzed in this layer. Moreover, the specifications of the fault test cases and the functional test cases are identified, including the test data and the expected output. Notably, the test cases are designed based on the test scenario using the ModelDesk tool. After test execution, the captured system behavior is compared with the desired behavior based on the defined functional and non-functional requirements to identify the violations. In addition, an analysis of the system’s response to the faulty components is performed in order to identify weaknesses in the system design.

Finally, in the last layer, two phases are considered, namely the system modeling and the environment visualization. As mentioned before, the ASM vehicle dynamics model from dSAPCE [52] is used as the target system from the automotive domain. The vehicle system has been modeled with high fidelity in the MATLAB/Simulink environment. However, the system model contains only the basic engine model. This limits the ability to capture and analyze detailed engine characteristics. Therefore, a complex ASM gasoline engine model was integrated into the dynamic model to address this challenge. To provide a comprehensive representation of the system behavior, other vehicle subsystems were also considered, including the electrical system, the drivetrain, the driver and the environment model. The code of all these models is generated and executed in the HIL simulator. On the other hand, the control algorithm is modeled separately and connected to the controlled system via a CAN bus model as a signal interface model. Notably, the FI framework is configured at this stage using the real-time interface CAN multimessage blockset (RTICANMM) model. Mathematically, the aerodynamic forces and moments can be represented as follows:

(3) $$F_{aero,i}=-\frac{1}{2}D_{air}S^{2}A_{i}\left(T\right)Z_{i}$$

(4) $$F_{aero,j}=-\frac{1}{2}D_{air}S^{2}A_{j}\left(T\right)Z_{i}$$

(5) $$F_{aero,k}=-\frac{1}{2}D_{air}S^{2}A_{k}\left(T\right)Z_{i}$$

(6) $$Tr_{aero,i}=-\frac{1}{2}D_{air}S^{2}A_{Mi}\left(T\right)Z_{i}Q_{ch}$$

(7) $$Tr_{aero,j}=-\frac{1}{2}D_{air}S^{2}A_{Mj}\left(T\right)Z_{i}Q_{ch}$$

(8) $$Tr_{aero,k}=-\frac{1}{2}D_{air}S^{2}A_{Mk}\left(T\right)Z_{i}Q_{ch}$$

(9) $$S=(S_{vehicle}-S_{wind})ij$$

where $S_{vehicle}$ and $S_{wind}$ are the vehicle and wind speeds [m/s], respectively. $D_{air}$ is the air density [kg/m^3^]. The vehicle’s longitudinal shadow area [m^2^] is represented by $Z_{i}$, while $D_{air}$ is the characteristic length for torque calculation [m]. A(T) represents the aerodynamic coefficients. Finally, *T* is the angle of incidence [deg].

Equation (10) represents the wheel speed $V_{wheel}$, where $Tr_{shaft}$, $Tr_{tire}$ and $Tr_{brake}$ represent the driving torque, effective tire torque and effective brake torque, respectively. $I_{wheel}$ is the moment of inertia about the wheel axis.

(10) $$I_{wheel}V_{wheel}=Tr_{shaft}+Tr_{tire}+Tr_{brake}$$

The motion for the movement of the steering rod can be represented mathematically as Equation (11):

(11) $$N_{st}q_{st}=F_{st,fl}+F_{st,fr}+F_{st,gear}+F_{st,fric}$$

where $F_{st,fl}$, $F_{st,fr}$, $F_{st,gear}$ and $F_{st,fric}$ represent the generalized force due to the front left tire forces, the generalized force due to the front right tire forces, the generalized force on the steering gear from the steering column and the generalized force due to friction in the steering rod, respectively. $N_{st}$ is the total mass along the axis of $q_{st}$ determined by the wheel inertia and masses.

At this stage, the driving environment is also modeled and visualized as a 3D visualization for the purpose of analyzing the effect of various environmental conditions on the system’s behavior. This is accomplished using the MotionDesk and ModelDesk tools. In addition to the normal condition, abnormal road and environmental conditions were also taken into account. For the environmental conditions, rain, snow, fog and lighting were considered. In particular, the road topology was modeled with surface irregularities to mimic the real environment to the greatest extent possible. Furthermore, the dynamic objects on the road were taken into account by modeling other vehicles and pedestrians. Finally, traffic signs and obstacles were modeled as transient events. To mimic the real-world road topology, a Google map of the target area served as the basis for the developed environment in our framework. In addition, the dimensions of the designed roads were set in the road geometry according to the standard dimensions of the real environment. Notably, the main goal of environmental modeling is to analyze the effects of environmental conditions, e.g., road and weather conditions, on the system behavior in a fault-free state and under fault conditions. The effects of FI on the system behavior during driving can be observed via 3D visualization.

## 4. Case Study and Experimental Implementation

Besides the platform’s hardware components, the software architecture of the case study used is also presented in this section. Additionally, the most important phases of the framework’s development process, including software and hardware communication, are presented.

### 4.1. Case Study Architecture

To demonstrate the capabilities and benefits of the proposed framework, a dSPACE dynamic vehicle system called ASM Vehicle dynamic with traffic was used. The case study was selected from the automotive domain for industrial automotive applications in the development phase.

**Figure 2:**
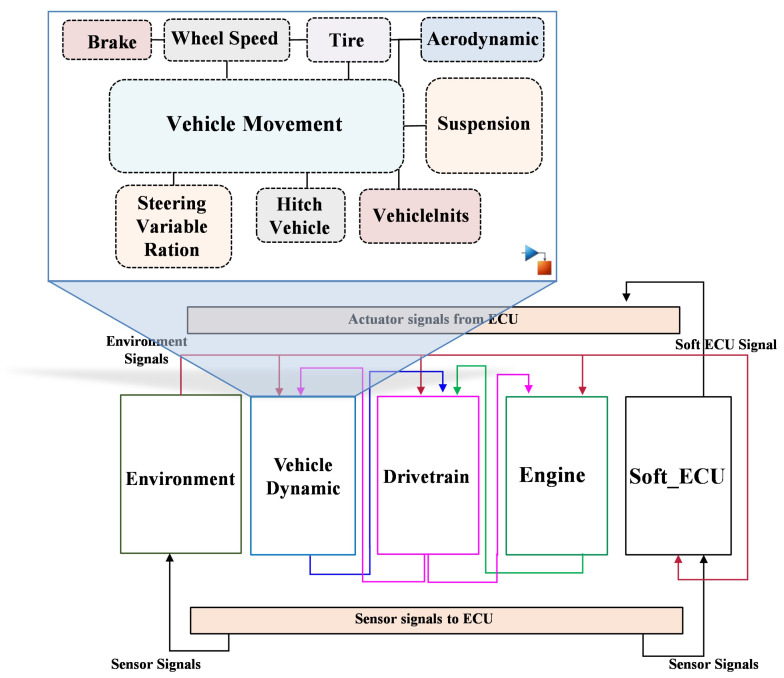
System architecture of the used case study.

The architecture of the selected vehicle dynamics system is shown in Figure 2. The MATLAB/Simulink environment was used to model and simulate the target system. By considering the detailed part of the system with a high degree of simulation fidelity, comprehensive characteristics of the system behavior can be captured. Several subsystems of the vehicle system have been considered, i.e., a basic gasoline engine, vehicle dynamics, powertrain and environment. However, due to the limitations of the basic engine model used in the case study, it was replaced by the ASM gasoline engine. Thus, two complex system models, vehicle dynamics and gasoline engine, can be integrated to obtain the detailed engine characteristics.

The vehicle dynamics system has been modeled in such a way that the dynamics of the vehicle can be simulated. This includes the longitudinal, lateral and vertical dynamics of the vehicle. The architecture of the mentioned model includes several subsystems, i.e., vehicle motion, aerodynamics, suspension, vehiclelnits, tires, wheel speed, brake, vehicle coupling and steering variable ration. Specifically, the brake model can simulate the pedal-to-torque transformation at each wheel. The suspension model is responsible for calculating the suspension kinematics and the suspension forces in the spring, shock absorber and stabilizer. The vehicle motion module calculates the vehicle motion and vertical wheel movements as a function of tire, aerodynamic, suspension, and mass forces and moments. The system parameters including the specifications of the model employed for real-time simulation in this study are summarized in Table 3.

Similarly, the gasoline engine system is designed on the basis of several subsystems, i.e., air path system, fuel system, piston engine system, exhaust system and cooling system. In addition to the above systems, the powertrain, driver and environmental electrical systems have been modeled, allowing the entire vehicle to be considered for real-time simulation. For each system in the vehicle, there is an ECU model that controls the corresponding system. Thus, during the development phase, high flexibility is achieved in selecting the SUT of any target system based on the applications. Note that, in our case, the gasoline engine ECU is selected as the SUT. Figure 2 shows the system architecture model of the selected case study, including the ECU and plant models. It is worth mentioning that this architecture allows two different modes, i.e., online and offline modes. Specifically, in the offline mode, i.e., using SoftECU, both the SUT model and the controlled model can be executed in the HIL simulator. On the other hand, in the online mode, the control system is executed in a separate target machine, while the controlled system is executed in the HIL simulator. In online mode, communication between the SUT and the plant is established via the CAN bus. In other words, the I/O blocks of the Real-Time Interface CAN Multimessage Block Set (RTICANMM) are used to model and simulate the signal interface. The RTICANMM is configured so that the baud rate of the CAN bus is set to 500 Kb/s. The list of CAN bus signals with their corresponding IDs is presented in Table 4.

**Figure 3:**
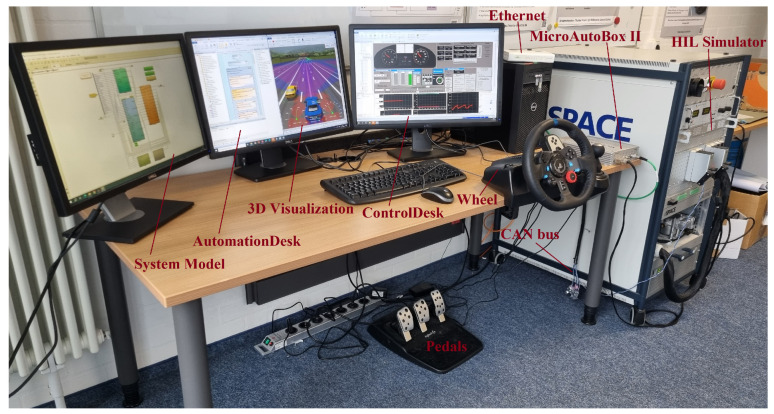
HIL real-time simulation-based virtual test drive.

Real-time simulation is performed by generating the ECU and vehicle model code and deploying it on the target machine. In this study, rapid control prototypes, i.e., a MicroAutoBox II embedded PC (DS1401 base board) with 900 MHz processor, 6th Gen.Intel^®^ CoreTM i7-6822EQ, 16 MB memory and 340 ms boot time for 3 MB application is used to mimic the functionality of the real ECU, while a SCAELXIO DS6001 processor board is used as the real-time processing hardware, as shown in Figure 3.

To evaluate the proposed approach, the runtime cost and the complexity in terms of creation and setup are specified. The maximum task execution time is 0.738 ms based on the debugging runtime information for a 10-second protocol and a scheduled sampling time of 1 ms. On the other hand, the execution time for each test case is 40,242.6164 ms, including the evaluation phase. The fact that three different fault attributes must be specified in the setup phase means that an unlimited number of possible configurations could be created, i.e., an unlimited fault space. The trade-off between high test coverage and finding the most effective critical faults is still a challenge for complex software systems. It is also worth noting that, along with the setup effort, a real-time system should be provided to ensure real-time execution of the system model along with the framework. In this study, three different factors were considered to determine the fault test cases. Among them, the first factor is the impact and speed of the injected fault that causes the termination of functionality, while the second factor is representative and realistic faults that are injected. Finally, the diversity of fault types and environmental conditions was the third factor.

### 4.2. Experimental Implementation

**Figure 4:**
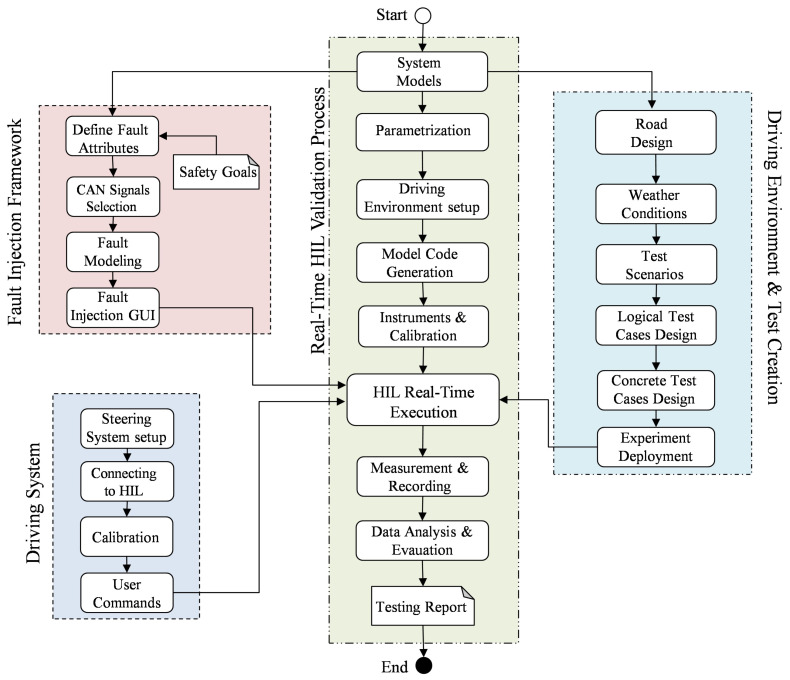
Flow chart of developing the proposed framework.

An overview of the main implementation phases is presented in this section. The development of the proposed framework is performed in four phases as shown in Figure 4. Specifically, the development of the FI framework, the configuration of the driving system, the test design, and the development of the driving environment are performed and linked to the main steps of the real-time HIL validation process. For this purpose, dSPACE software tools (version release 2021-B) are used, including ModelDesk (version 5.6), MotionDesk (version 4.9) and ControlDesk (version 7.5) [53].

#### 4.2.1. Real-Time Automated Fault Injection Framework

According to the ISO 26262 standard, the FI method is highly recommended to validate the safety and reliability properties at different stages of the system development process. It is also considered a powerful method for evaluating the robustness of fault-tolerant systems in the presence of faults. The core concept of the method is to introduce faults into the SUT and evaluate its response to the abnormal conditions.

Three different attributes should be defined before the FI experiment is performed, i.e., fault type, fault location and FI time. In particular, the fault types are usually identified based on the test object and the objective. The focus of this study is on the fault types that occur in the hardware components, i.e., sensor and actuators. Gain, offset, hard-over, stuck-at, delay, noise, packet loss, drift and spike faults are the main types that fall under the category of sequential data-related faults. Fault location, as the second FI attribute, is selected based on the list of system signals that represent the potential fault location. As stated in [13], sensor and actuator components are inevitably at risk of failure. In addition, ECUs, networks, gateways, a power supply, vehicle subsystems and a data acquisition system are also potential fault locations in the automotive system. The third attribute, FI time and duration, is specified based on the interest period from the driving scenario when the system state should be evaluated, e.g., the acceleration, braking or deceleration periods of the vehicle. In this study, and based on the safety goals document, the aforementioned attributes are defined, which serve as a basis for the following implementation phases.

It is worth noting that, in the system integration test phase of the V-model, i.e., ISO 26262-4, the real-time constraints of the system execution in the target machine should be ensured. Therefore, in this work, the FI process is performed at the signal interface between the SUT (real ECU) and the HIL simulator. Thus, based on the documented fault attributes, the system signals to be manipulated as fault locations are accessed and selected via the CAN bus network. This allows the target system variables to be manipulated programmatically based on the accessed signals in the CAN bus network. This, in turn, ensures the black-box implementation of fault modes without the need to extend the system architecture model.

**Figure 5:**
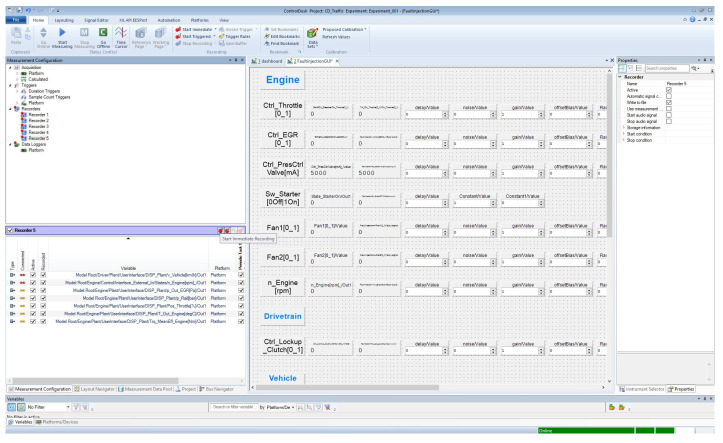
GUI of the real-time fault injection.

Thanks to the FI GUI (Figure 5), FI experiment configurations can be modified and specified by the user before and during real-time execution according to the test requirements and objectives. It is worth mentioning that the developed framework allows injecting faults individually or simultaneously. The majority of the critical system components were covered in this study, including various vehicle subsystems, such as the engine, vehicle dynamics and powertrain. Furthermore, it supports the analysis of the effect of transient and permanent faults on the system behavior in real time. Finally, two execution modes are provided by the framework, i.e., automated and manual execution. If the test drive is performed automatically by the machine, the faults can be injected automatically using an automation software tool, i.e., AutomationDesk (version 6.6). In this case, the execution process follows a sequence of block hierarchies with three phases, i.e., reading, manipulating signals and writing [54]. In the user-based virtual test drive, on the other hand, faults are injected manually during real-time HIL execution.

#### 4.2.2. Steering System for Manual Driving

Real Logitech steering wheel, gearshift and pedals are used for manual driving. The driving elements are connected to the host PC via USB communication protocol. Before the driving elements are connected to the HIL system, the PC is used for setup and configuration to define the devices. After setup and configuration, the driving system is connected to the HIL simulation via the ControlDesk tool, which connects the software steering controller to the real physical steering wheel. By using the aforementioned controller, not only can the variable values of the device be adjusted, but also its range can be calibrated according to the experimental requirements. Once the steering device is connected, the calibration process with the real-time simulator takes place. In this phase, the “Angle of Steering” variable is manually calibrated to match the 3D virtual driving environment; in this study, the range is specified to be [300–300]. Next, the pedal variables, i.e., Pos_ACC, Pos Brake and Pos_Clutch are connected and calibrated according to the acceleration, braking and clutch position with a range of [0–100]. Finally, the gear variables are set in ControlDesk so that each gear level is set to a specific number as an on/off button in the steering controller. During the manual test drive, the driving commands are received from the driving system as external inputs to the systems according to the user’s actions. Then, in online mode, the vehicle system executes the driving scenario during real-time simulation, taking into account the user’s driving commands.

#### 4.2.3. Driving Environment and Test Creation

**Figure 6:**
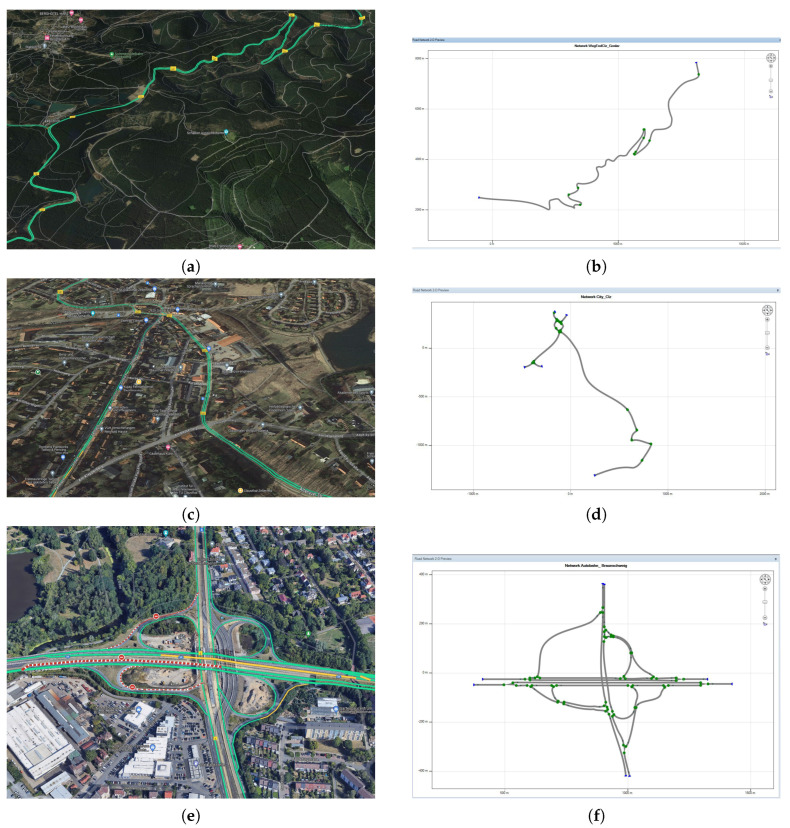
Road network topology and traffic design. (**a**) Real non−urban area (CLZ−GS). (**b**) Designed non-urban area (CLZ−GS). (**c**) Real urban area (CLZ). (**d**) Designed urban area (CLZ). (**e**) Real highway area (BS). (**f**) Designed highway area (BS).

Once the system models have been selected and imported, the test environment and test scenarios are designed and created. In addition to configuring the vehicle, ModelDesk allows creating the test environment, i.e., the road, driving maneuvers and traffic. In particular, the road networks can be created with their characteristics, e.g., lanes, lines and traffic signs, intersections, height, inclination and surface. In our proposed approach, the test scenarios and environmental conditions have been designed to mimic real-world situations. To this end, the road topology of the critical area in our city, i.e., the non-urban stretch between Goslar and Clausthal-Zellerfeld, was taken into account. Beyond that, the most critical weather scenarios affecting the driving behavior were modeled according to the real environmental conditions in this area. In this study, three areas have been modeled to capture different conditions within and between the cities. The first section considered is the route between ISSE and ZOB within the city of Clausthal-Zellerfeld (CLZ). By doing so, the validation process of the system considering the road and environmental characteristics within urban areas is enabled. Some examples of the the situations considered during the modeling are the pedestrians, the traffic lights and traffic with limited speed. The second area covers the non-urban roads, i.e., the road characteristics between the CLZ and the city of Goslar. Due to the fact that the mentioned direction is a mountain road, different road characteristics were considered in terms of road curvature and slope. In addition, the mentioned road has two directions of vehicle traffic. Finally, the third area includes part of the highway and the entrance to the city of Braunschweig. Thus, most of the normal and abnormal conditions have been covered during the environment design phase, i.e., non-urban, within the urban area and on the highway, as shown in Figure 6b,d,f, respectively.

**Figure 7:**
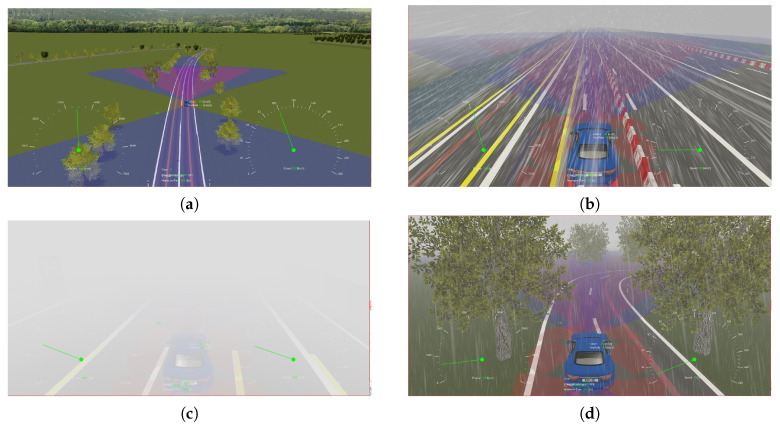
Testing environment under various environmental conditions. (**a**) Sunny weather. (**b**) Rainy weather. (**c**) Snowy weather. (**d**) Foggy weather.

The next phase is to develop the 3D visualization of the testing environment including different weather conditions. To this end, the capabilities of the MotionDesk tool have been employed. Four environmental conditions were considered, i.e., sunny, rainy, snowy and foggy, as shown in Figure 7a–d, respectively. In addition, both day and night vision have been considered in the visualization process. Notably, the blue color in the Figure 7 represents the area of sensor scanning.

To define the motion of dynamic objects in the driving environment, such as vehicles and pedestrians, ModelDesk is used. Several normal and abnormal conditions were considered. Among them, the obstacle and construction area were covered to analyze and validate the system behavior in unexpected conditions. Notably, in the proposed study, obstacle detection/avoidance is performed using the functions of the ModelDesk and MotionDesk software tools from dSPACE. The focus of the study, however, is on analyzing the internal system errors in the internal components, taking into account the environmental conditions.

Several test cases are created from the test scenario. These include the input data, the expected output and the evaluation functions. The requirements-based testing method is basically used to design and implement the test cases so that the functionality of the SUT is verified against the requirements specifications. Therefore, the test cases are generated from the ECU requirements to ensure a high level of test coverage. In general, the test cases in these tests have a consistent structure and procedure so that an expected output is compared with the actual output of the SUT. The results can be evaluated as pass or fail depending on the execution of the test cases. Notably, an automation software tool, e.g., AutomationDesk is used to automatically design, execute and evaluate a large number of test cases. Once the test environment, test scenarios and test cases have been developed, the generated experiment data are transferred to the HIL simulator for real-time execution.

#### 4.2.4. Real-Time HIL Validation Process

Due to its vital role in providing a safe, flexible, reliable and realistic simulation platform, HIL simulation has been explicitly recommended by the ISO 26262 standard to perform various testing methods during the development process. However, before performing the test activities, the test platform configurations should be specified. As shown in Figure 4, the validation process starts by importing the system models, i.e., the SUT and the controlled plant model, from the development phase. The system parameters are set using ModelDesk to specify the static system variables, known as the parameterization process. In this study, the model-based design approach used during the development of automotive software systems is considered. Based on this approach, the code can be automatically generated from the system model using software tools. Thanks to the simulation tools, i.e., Simulink with ConfigurationDesk, the system model code can be automatically generated and deployed on the target machine. In this case, the object code of the SUT is deployed and executed in the real ECU. On the other hand, the executable object code of the controlled system model is executed in the HIL simulator, including the RTICANMM between the ECU and the HIL simulator.

Once the generated model code and the developed test environment are deployed on the target machine, the software application can be executed on the target machine using ControlDesk’s layout instrumentation. Thanks to the synchronization between the software tools used, the real-time system execution is visualized as an online 3D animation in MotionDesk, which simultaneously shows the system behavior within the test environment. In other words, the motion of the target system and its interaction with other dynamic objects in the 3D world are visualized. In particular, the manual driving mode allows the user to perform the driving scenario with full control of the driving functions. Specifically, the driving commands are received from the external driving device which is controlled by the user. Unlike automated driving, manual driving does not require the user to follow a pre-defined routine, but instead allows the user to create his or her own scenario based on human–machine interaction within the developed test environment. During online execution and based on user-configured FI attributes, faults are injected into the accessed system signals over the CAN bus network between the ECU and the HIL simulator.

In addition to controller calibration and diagnostics, ControlDesk provides a measurement and data recording capability. After specifying the recording system parameters, e.g., sampling time and target signals, the selected system signals are recorded as time series data during the real-time simulation.

In the evaluation phase, i.e., in the automated driving mode, the recorded system behavior is evaluated against the expected behavior using an evaluation function. Based on the defined evaluation criteria, any deviation of the target signals from the expected values out of tolerance is considered a failure. Thus, in case of the failure occurrence, the executed test cases are evaluated as failed. Otherwise, the test results are evaluated as passed if the actual measured signals are within the expected range. On the other hand, as a result of manual driving, uncertainty data including noise are recorded. In this case, any abnormal behavior detected by the test driver is flagged as a potential failure. The corresponding recorded data set of abnormal behavior is analyzed by the tester against the requirements of the intended performance and the safety criteria. Notably, the analysis process of the recordings is performed manually based on expert knowledge to identify the nature and location of the detected fault. Finally, as a result of the test, which is evaluated in both driving modes, the test report is generated, containing all findings, observations and evaluation results. The report is then sent back to the development team to resolve and mitigate the identified defects.

## 5. Results and Discussion

In this section, the evaluation results of the proposed framework are presented and discussed. Focusing on three different test environments, i.e., non-urban, urban and highway, the system behavior under different internal and external conditions is analyzed. Based on the mentioned test scenarios, as an external abnormal condition, the system validation process under different environmental conditions is demonstrated. In addition, as an internal abnormal condition, the validation process of the target system under single and concurrent faults are discussed.

### 5.1. Validation of the Real-Time Simulation System

**Figure 8:**
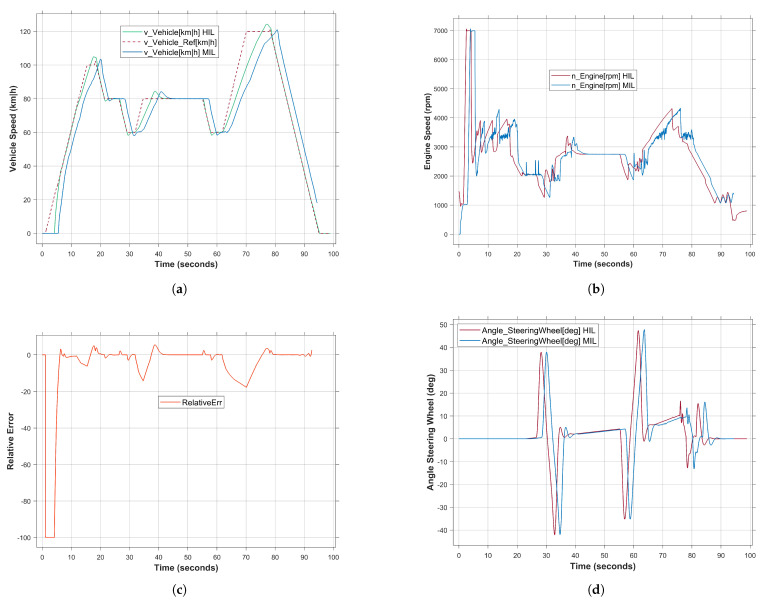

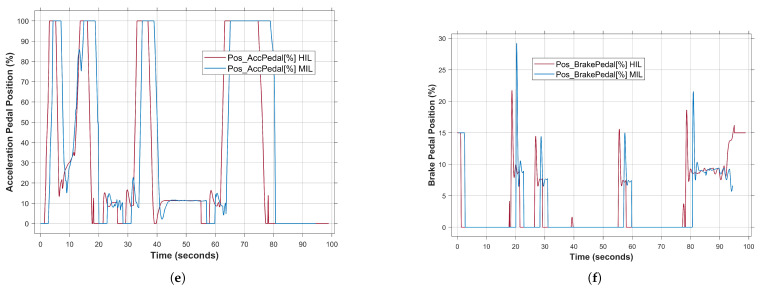
Comparison results of the real-time HIL and non-real-time MIL simulation. (**a**) Vehicle speed over the driving time. (**b**) Engine speed over the driving time. (**c**) Relative error of the system performance in terms of vehicle speed. (**d**) Angle steering position. (**e**) Acceleration pedal position. (**f**) Brake pedal position.

To verify the effectiveness of the proposed framework, the results of the real-time simulation of the HIL system were compared with the non-real-time simulation of MATLAB/Simulink (MIL). To this end, the RoadWork_Highway scenario was conducted at both test levels mentioned, i.e., MIL and HIL, with the same test specifications. The desired theoretical behavior was identified as a reference for comparing the simulation results, as shown in Figure 8a, curve with brown color. The test scenario applied mimics a driving situation on the highway where a construction site is encountered. The vehicle speed should reach two maximum values at 15 s and 70 s with 100 and 120 (km/h), respectively. However, according to the vehicle speed reference profile, the behavior of the vehicle system changes so that the vehicle speed remains in the range [100–60] during the period [19–65] s, while passing through the work zone on the highway. In Figure 8a, it can be clearly noticed that the system behavior with the proposed framework is closer to the decided behavior compared to the MIL results. The reason behind this fact is that the control strategy in HIL is executed accurately considering the real-time constraints. This in turn leads to a smooth change in the system state with little variation in the engine speed (rpm) compared to the MIL results (see Figure 8b). To calculate the relative error, the discrepancy between the theoretically desired behavior and the actual real-time simulation results of the HIL was calculated. Figure 8c shows the calculated error with low-level deviation while driving in the construction zone. The maximum relative error value is 13 at 34.8 s and the average value is 2.52. However, the absolute error value increases to 17.6 at 70 s due to the sudden change in the system speed from 60 to 120 km/h. Nevertheless, compared to the MIL results, the system performance with the HIL system is more accurate in performing the desired behavior.

As a comparison between MIL and HIL results, Figure 8d–f show the behavior of the driving elements, i.e., the angle of the steering wheel, the position of the accelerator pedal and the position of the brake pedal. It can be observed that both simulation results show similar behavior, with the exception that the HIL system is able to show a more accurate behavior with less fluctuations, especially in the case of driving between 20 and 62 s (Figure 8e). Similarly, Figure 8f shows the status of the brake pedal with accurate response to the control command during the driving time. It can be concluded that using the HIL system as a platform to perform the virtual test drive has high accuracy and lower relative errors compared to non-real-time simulation methods.

To demonstrate the superiority of the proposed framework compared to other approaches, the characteristics of our approach in terms of relative error, fault injection capability and real-time constraint consideration have been summarized in Table 5. It can be concluded that our proposed approach has a lower error rate compared to related works. Besides the real-time constraint consideration during the simulation process, the ability to analyze the system under faulty components shows its superiority and applicability compared to the related proposed studies.

### 5.2. System Validation under Various Roads and Weather Conditions

**Figure 9:**
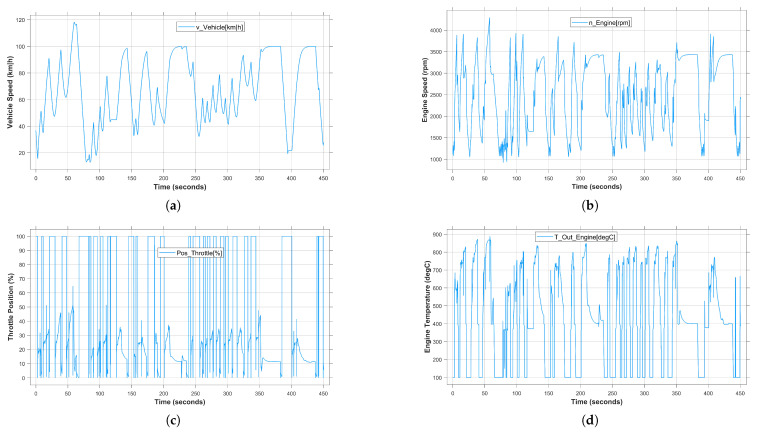
Vehicle system behavior during a real-time virtual test drive. (**a**) Vehicle speed. (**b**) Engine speed. (**c**) Throttle position. (**d**) Engine temperature.

The consideration of abnormal road conditions in the driving environment, e.g., mountain roads with steep slopes and curves, plays an important role in the analysis of the system behavior in critical situations, especially for safety-critical systems. Therefore, in this study, the characteristics of the non-urban road between Goslar and Clausthal city have been modeled so that the unexpected faults and unobserved problems can be detected. Figure 9 shows the vehicle system behavior during a virtual test drive performed manually by the user in the real-time for 450 s. Specifically, the recorded system variables, i.e., vehicle speed, engine speed, throttle position and engine temperature are shown in Figure 9a–d, respectively. The above scenario was performed under fault-free conditions. As can be observed, the more curves in the road, the more the system’s dynamic behavior changes. Due to the mentioned conditions, the vehicle speed changes as a multi-mode behavior between 20 and 100 km/h without being able to maintain a steady state. Specifically, the vehicle speed decreases significantly from 100 to 40 km/h to tackle and skip turns. The corresponding engine speed, throttle position and engine temperature change frequently depending on the scenario. However, it should be noted that the more complex the system behavior, the more difficult the analysis and fault detection process.

**Figure 10:**
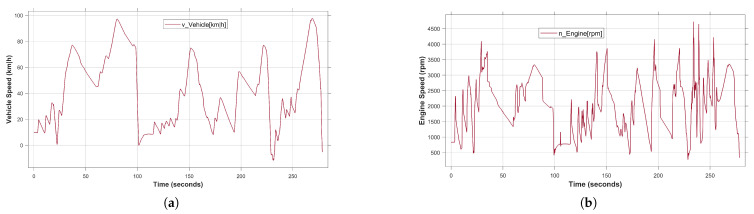
User-based driving behavior under foggy weather condition. (**a**) Vehicle speed under abnormal vision condition. (**b**) Engine speed under abnormal vision condition.

Driving in snowy and foggy conditions is considered to be a risk not only at night but during the day as well. Therefore, the consideration of various weather situations is essential for the safety validation process. Aiming to overcome the limitations of real-world weather testing in terms of cost and safety, the proposed framework has covered the most critical situation of the testing environment. In this study, in addition to sunny weather, the developed system has been validated under rainy, foggy and snowy weather. During manual driving in the above cases, the user is unable to drive smoothly due to blurred vision. The effect of the aforementioned weather conditions on the user-based driving behavior is shown in Figure 10. Compared to the fault-free behavior shown in Figure 9a, the vehicle speed in Figure 9a changes slightly between 0–50 s and 100–150 s. Similarly, the engine speed in Figure 10a follows an uncertain pattern with dynamic nonlinear behavior.

### 5.3. System Validation in Occurrence of Single Faults

Unexpected faults in sensors and actuators within complex systems can potentially propagate to different components and subsystems. This phenomenon is known as fault propagation [56]. This propagation can have a significant impact on the proper functioning of the system. For vehicle functions, especially ADAS and Autonomous Driving, failures due to fault propagation pose a violation to safety objectives and have serious consequences. To address these risks, it is essential to have simulations that reproduce the behavior of the system in the case of fault occurrence. Performing these simulations allows in-depth analysis of the nature and causes of the safety risks associated with fault propagation.

**Figure 11:**
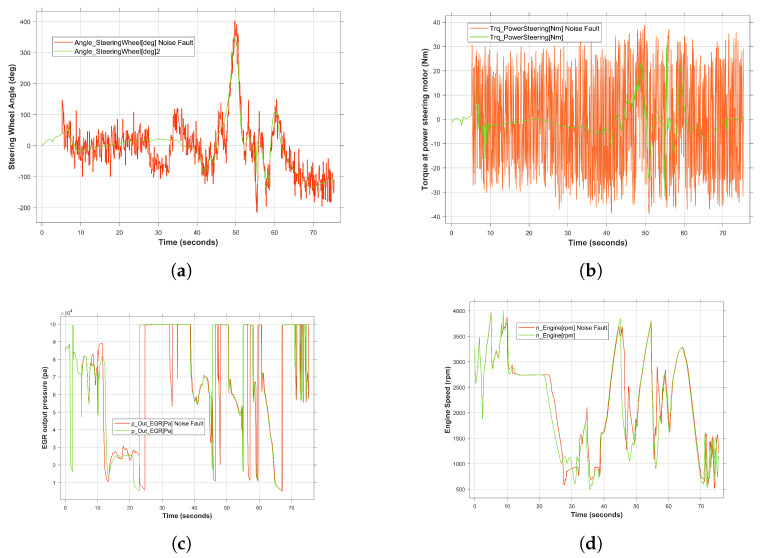
The effect of fault propagation on system behavior under single fault condition in steering wheel angle. (**a**) Noise fault injection in the signal of steering wheel angle sensor. (**b**) Torque at power steering motor under propagated noise fault. (**c**) EGR output pressure under propagated noise fault. (**d**) Engine speed under propagated noise fault.

**Figure 12:**
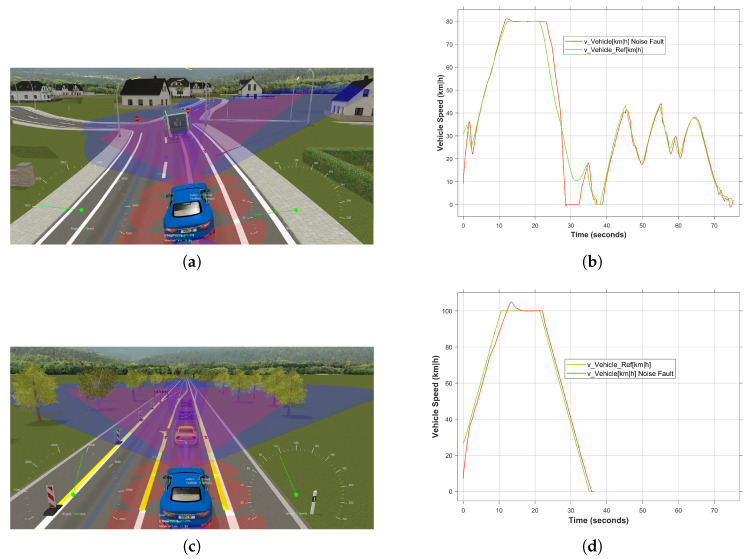
The effect of different road and environmental conditions on FI experiments. (**a**) Three-dimensional visualization of CLZ driving scenario. (**b**) Vehicle speed in the occurrence of noise fault. (**c**) Three-dimensional visualization of Road_Work driving scenario. (**d**) Vehicle speed behavior under noise fault.

In the proposed framework, nine different types of time-series related faults [57] have been considered. Among them, the effect of injecting the noise fault into the steering wheel angle sensor has been demonstrated in Figure 11. The reason for selecting this sensor as the fault injection location is its critical effect on the safety-related automotive system, i.e., the steering system. The fault was introduced at 5 s, when the urban driving scenario, namely CLZ, was selected. The fault injected at the component level in the steering wheel angle sensor signal (Figure 11a) has propagated to the subsystem level, resulting in an error in the torque signal at the power steering motor (Figure 11b). This, in turn, has led to a failure at the interface of the vehicle dynamic subsystem. The aforementioned failure propagated and resulted in an error in the engine subsystem, i.e., in the EGR output pressure, (Figure 11c). Compared to the fault-free behavior, i.e., green curve, in Figure 11d, the propagated fault caused a deviation from the desired behavior in the period [5 to 60] s. By increasing the ratio of the deviation from 22 s, the system becomes unable to follow the defined scenario. Consequently, the fault propagates from the sensor to other system components, resulting in the termination of the required functionality at the system level after 28 s (Figure 12b). However, from 40 s, since the target system is capable of performing the intended functionality, the resulting deviation is considered to be an acceptable anomaly that does not pose a serious risk. The reason behind this fact is that the SUT mitigates the injected fault during this period to achieve the desired behavior.

Thus, the analysis process can be repeated by changing one or more of the configurations of the injected faults, i.e., type, location or time. By analyzing the system behavior under fault conditions during a virtual test drive, it is possible to identify the weak points within the system and improve the safety mechanisms. This, in turn, leads to the improvement of the safety aspects of the target system. As a result, not only can potential hazards be averted, but the intended functionality of the system can be maintained, ensuring overall safety.

To illustrate the impact of the driving scenario on the FI experiment, the aforementioned FI results, i.e., with the CLZ scenario (Figure 12a), have been compared with those of another driving scenario, namely the basic Road_work scenario (Figure 12c). In this regard, the same FI configurations, including type, location and time, have been considered in both experiments. As illustrated in Figure 12d, in the case that the desired system behavior is not complex, the SUT is capable of mitigating the fault and performing the required functionality. Conversely, due to the dynamic behavior required within the city (Figure 12b), the effect of the fault is observable and poses a risk.

### 5.4. System Validation in Occurrence of Concurrent Faults

According to ISO 26262, not only a single fault, but also the simultaneous occurrence of two faults at different locations could violate the safety objectives. Due to the fact that two faults contribute to the resulting system-level behavior, the analysis process is considered critical and challenging during testing. The integrated FI method in the proposed approach provides the ability to inject concurrent faults into the target components in real time to analyze the safety and reliability characteristics.

**Figure 13:**
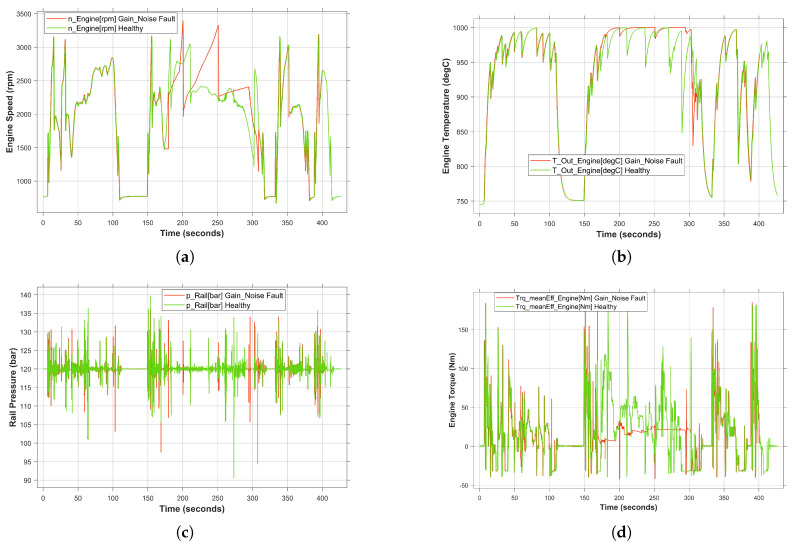
System behavior under simultaneous faults condition. (**a**) Engine speed under gain and noise faults. (**b**) Engine temperature under gain and noise faults. (**c**) Rail pressure under gain and noise faults. (**d**) Engine torque under gain and noise faults.

Due to the fact that the occurrence of simultaneous transient faults is more critical than permanent faults, in this study, the effect of two sensor-related faults on the system behavior has been demonstrated. Specifically, gain and noise faults were simultaneously injected into the accelerator pedal sensor and RPM sensor, respectively. Both faults have been activated from 170 to 330 s as shown in Figure 13. Once the faults have been injected, the deviation of the system behavior from the healthy behavior (green curve) can be clearly observed in a red curve. In this case, the mitigation of the concurrent faults by the SUT is considered as a complicating factor. Each fault played an individual role in shaping the erroneous system behavior. This, in turn, caused a temporary cessation in the execution of the intended functionality. Specifically, as shown in Figure 13d, the engine is unable to provide the required torque to the drive system under fault conditions. Similarly, the engine temperature and rail pressure were also affected by the failure, as shown in Figure 13b and Figure 13c, respectively.

In terms of vehicle speed, the aforementioned functionality termination caused an observable change in vehicle behavior. However, the deactivation of the fault at 330 s allows the SUT to return to the safe state, resulting in the desired behavior. Thus, the critical concurrent faults with their corresponding attributes, type, location and time, can be identified in an efficient manner. However, the larger the set of the parameters, the larger the number of experiments that can be performed to achieve a high degree of test coverage. Therefore, to solve this problem, an automation tool has been integrated into the proposed framework so that the injection process is performed automatically. Thus, much of the analysis process can be carried out according to the safety requirements for validation.

## 6. Conclusions

A novel real-time testing framework for the validation of automotive software systems, i.e., safety-related systems, is proposed in this article. The main objective of the proposed approach is to perform the functionality and safety validation process at the system integration phase of the V-model. Based on HIL real-time simulation, a virtual test drive is enabled with automated and manual driving mode, considering the user behavior. In contrast to the traditional simulation methods, the proposed work allows not only the validation process in fault-free mode, but also the analysis of the system behavior under fault occurrence. Both single and simultaneous sensor/actuator-related faults can be injected during the real-time execution of the system in the target machine. Specifically, gain, offset/bias, noise, hard-over, spike, stuck-at, packet loss, delay and drift faults have been considered. These types of faults can be injected manually or automatically, as permanent or transient faults. In addition to traffic and road conditions, various weather conditions have been covered and included in the developed framework. Specifically, the system behavior can be validated under sunny, rainy, foggy and snowy weather conditions. Compared with the non-real-time simulation methods, the evaluation results show that the system behavior exhibits high performance in terms of accuracy with an average relative error of 2.52. Moreover, the comparison study with the related work demonstrated the superiority of the proposed approach with high capability of real-time simulation under critical situations. All in all, the proposed framework allows performing the validation activities according to the ISO 26262 standard to ensure safety and functionality during the development of safety critical systems. This, in turn, not only contributes to improving the safety and reliability of the developed system, but also reduces the cost and effort of real-world testing.

As future work, it is planned to extend the features and capabilities of the proposed approach by integrating real vehicle components and subsystems into the framework, e.g., real fuel pressure sensors and throttle valve. In this way, the uncertainty of the data caused by real elements will be taken into account during the validation process under realistic real-time conditions. However, this requires additional effort and cost in terms of computation time and setup. Moreover, to efficiently analyze the generated time series test data sets, AI-based intelligent models can be integrated into the proposed approach to automatically detect and classify the defects.

## Figures and Tables

**Table 1:** Overview of the related work.

Reference	Application Domain	Contributions	Driving Mode	Testing Level	Driving Environment Conditions	Faulty Conditions	Remarks
[34]	Safety analysis of autonomous vehicle	Simulation framework	Predefined virtual driving scenarios	MIL	Traffic-relevant objects	Single fault of sensors	Reliability: low Cost: low Safety: high Scene simulation: no limits
[35]	Control algorithm validation of automated vehicles	Simulation methodology	Predefined virtual driving scenarios	MIL	Road topology and surface unevenness	Not considered	Reliability: low Cost: low Safety: high Scene simulation: no limits
[36]	Testing of autonomous vehicles	Simulation toolchain	Variable virtual driving scenarios	SIL and MIL	Driving scenario dynamics, objects and environmental conditions	Not considered	Reliability: low Cost: low Safety: high Scene simulation: no limits
[38]	Testing ADAS	Modular topology	Real-world driving	VIL	Surrounding vehicles, pedestrians and road lines	Not considered	Reliability: high Cost: high Safety: low Scene simulation: high limits
[40]	Test and verify ADAS	Testing methodology	Real-world driving	VIL	Traffic, vehicle sensor and static environment	Not considered	Reliability: high Cost: high Safety: low Scene simulation: high limits
[45]	Validation of motion controllers of electric vehicles	Experimental platform	Manual driving in lab	HIL	Road conditions	Not considered	Reliability: high Cost: low Safety: high Scene simulation: low limits
[46]	Testing ECU of autonomous vehicle	Simulation platform	Self-driving	HIL	Multi-agent interaction and environment building	Not considered	Reliability: high Cost: low Safety: high Scene simulation: low limits
[47]	Evaluate ESC control units	Test bench	Virtual driving scenarios	HIL	Vehicle visualization	Not considered	Reliability: high Cost: low Safety: high Scene simulation: low limits
[48]	Evaluation of cooperative eco-driving systems	Integrated driving simulator	Manual driving	HIL	Traffic visualization	Not considered	Reliability: high Cost: low Safety: high Scenario simulation: low limits
Proposed Model	Real-time validation of ASSs	Real-time virtual testing framework	Manual driving and self-driving	SIL, HIL	Environmental conditions, moving objects, traffic obstructions and infrastructure, road topology	Single and simultaneous faults	Reliability: high Cost: low Safety: high Scene simulation: low limits

**Table 2:** Value of $g_{v}$ and $s_{v}$ for all fault types.

Fault Type	($g_{v}$) Value	($s_{v}$) Value
Healthy Signal	1	0
Stuck-at Fault	0	0 or 1, and it varies on time
Offset/Bias Fault	1	fixed constant value
Gain Fault	Greater than 1	0
Noise Fault	1	random value
Hard-Over Fault	0	higher than maximum threshold
Spike Fault	1	value varies on time
Drift Fault	1	value increases on time
Packet Loss Fault	0	0
Delay Time Fault	0	last cycle value of h(t) based on time given

**Table 3:** System parameters of the used case study for real-time simulation.

Parameter	Unit	Value
Mass vehicle with wheel	kg	1880
Density air	kg/m^3^	1.188
Mass front wheel	kg	35
Mass left wheel	kg	32
Wheel base	m	2.9742
Inertia about the wheel ration	kgm^2^	1.5
Engine speed setpoint during start up	rpm	1100
Engine displacement	cc	2900
Engine power	kW	185
Car type	-	MidSizeCar
Transmission type	-	Stepped Automatic
Tire model	-	Magic Formula

**Table 4:** List of the CAN bus signals and their identifiers.

CAN Bus Signals	Unit	ID
Pos_Throttle	[%]	26
p_InMan_Rel	[Pa]	31
p_ExhMan_Rel	[Pa]	34
T_Out_Turb_HP	[degC]	36
n_Engine	[rpm]	1
p_Out_Airfilter_Rel	[Pa]	6
p_In_Throttle_Rel	[Pa]	7
Ctrl_Throttle	[0_1]	25
omega_TC	[rpm]	8
Angle_SteeringWheel	[deg]	15
omega_TC_HP	[rpm]	16
Trq_MeanInd_Engine	[Nm]	93
T_In_InterCooler	[degC]	23
T_Out_InterCooler	[degC]	24
q_Mean_Inj	[mm^3^|cyc]	2
Trq_MeanEff_Engine	[Nm]	3
Pos_AccPedal	[%]	4
Pos_BrakePedal	[%]	5
Ctrl_FuelMeterUnit	[mA]	50
T_Water	[degC]	47
p_Rail	[bar]	48
EGRRate	[%]	30

**Table 5:** Comparison between the results of the proposed method and other related works.

Reference	Average Error	Fault Injection	Real-Time Constraints
[38]	4	Not Considered	Considered
[47]	8	Not Considered	Considered
[55]	-	Not Considered	Considered
[39]	5	Not Considered	Considered
[48]	-	Not Considered	Considered
Proposed work	2.52	Considered	Considered

## Data Availability

Data available on request due to restrictions.

## References

[1] Vogelsang A. (2020). Feature dependencies in automotive software systems: Extent, awareness, and refactoring. J. Syst. Softw..

[2] Kukkala V.K., Tunnell J., Pasricha S., Bradley T. (2018). Advanced driver-assistance systems: A path toward autonomous vehicles. IEEE Consum. Electron. Mag..

[3] Ebert C., Favaro J. (2017). Automotive software. IEEE Softw..

[4] Bello L.L., Mariani R., Mubeen S., Saponara S. (2018). Recent advances and trends in on-board embedded and networked automotive systems. IEEE Trans. Ind. Inform..

[5] (2018). Road Vehicles Functional Safety.

[6] Rana R., Staron M., Berger C., Hansson J., Nilsson M., Törner F. Increasing efficiency of iso 26262 verification and validation by combining fault injection and mutation testing with model based development. Proceedings of the International Conference on Software Engineering and Applications.

[7] Samuel S., Austin L., Morrey D. (2002). Automotive test drive cycles for emission measurement and real-world emission levels-a review. Proc. Inst. Mech. Eng. Part J. Automob. Eng..

[8] Krämer M., Röhringer A., Kirchner W., Vogel T., Rochlitzer J., Heiniger B. (2009). Programme Management and Project Control. ATZextra Worldw..

[9] Yu C.H., Chen Y.Z., Kuo I.C. The benefit of simulation test application on the development of autonomous driving system. Proceedings of the 2020 International Automatic Control Conference (CACS).

[10] Szalay Z., Szalai M., Tóth B., Tettamanti T., Tihanyi V. Proof of concept for Scenario-in-the-Loop (SciL) testing for autonomous vehicle technology. Proceedings of the 2019 IEEE International Conference on Connected Vehicles and Expo (ICCVE).

[11] Stahl T., Betz J. An open-source scenario architect for autonomous vehicles. Proceedings of the 2020 Fifteenth International Conference on Ecological Vehicles and Renewable Energies (EVER).

[12] Gietelink O., Ploeg J., De Schutter B., Verhaegen M. (2009). Development of a driver information and warning system with vehicle hardware-in-the-loop simulations. Mechatronics.

[13] Theissler A. (2017). Detecting known and unknown faults in automotive systems using ensemble-based anomaly detection. Knowl.-Based Syst..

[14] Nair V.V., Koustubh B.P. Data analysis techniques for fault detection in hybrid/electric vehicles. Proceedings of the 2017 IEEE Transportation Electrification Conference (ITEC-India).

[15] Safar M., El-Moursy M.A., Abdelsalam M., Bakr A., Khalil K., Salem A. (2019). Virtual verification and validation of automotive system. J. Circuits Syst. Comput..

[16] Tibba G., Malz C., Stoermer C., Nagarajan N., Zhang L., Chakraborty S. Testing automotive embedded systems under X-in-the-loop setups. Proceedings of the 2016 IEEE/ACM International Conference on Computer-Aided Design (ICCAD).

[17] Plummer A.R. (2006). Model-in-the-loop testing. Proc. Inst. Mech. Eng. Part J. Syst. Control Eng..

[18] Bittar A., Figuereido H.V., Guimaraes P.A., Mendes A.C. Guidance software-in-the-loop simulation using x-plane and simulink for uavs. Proceedings of the 2014 International Conference on Unmanned Aircraft Systems (ICUAS).

[19] Mina J., Flores Z., López E., Pérez A., Calleja J.H. Processor-in-the-loop and hardware-in-the-loop simulation of electric systems based in FPGA. Proceedings of the 2016 13th International Conference on Power Electronics (CIEP).

[20] Isermann R., Schaffnit J., Sinsel S. (1999). Hardware-in-the-loop simulation for the design and testing of engine-control systems. Control Eng. Pract..

[21] Bokc T., Maurer M., Farber G. Validation of the vehicle in the loop (vil); a milestone for the simulation of driver assistance systems. Proceedings of the 2007 IEEE Intelligent Vehicles Symposium.

[22] Garousi V., Felderer M., Karapıçak Ç.M., Yılmaz U. (2018). Testing embedded software: A survey of the literature. Inf. Softw. Technol..

[23] Simulink. MathWorks. https://www.mathworks.com/products/simulink.html.

[24] Pintard L., Fabre J.C., Kanoun K., Leeman M., Roy M. (2013). Fault injection in the automotive standard ISO 26262: An initial approach. Proceedings of the European Workshop on Dependable Computing.

[25] Oberst J., Kemper H., Günther A., Schick B. (2024). Consistent Evaluation of Automated Driving Functions Based on Vehicle-in-the-Loop. ATZ Worldw..

[26] Kim Y., Do Na S., Park P., Lim J., Kyeong J. (2024). A Study on the Development of Architecture Virtual Driving Performance Using Concept Model.

[27] Reick B., Pintaric I., Osorio C. (2023). HIL Based Real-Time Co-Simulation for BEV Fault Injection Testing.

[28] Wynne R.A., Beanland V., Salmon P.M. (2019). Systematic review of driving simulator validation studies. Saf. Sci..

[29] Bruck L., Haycock B., Emadi A. (2020). A review of driving simulation technology and applications. IEEE Open J. Veh. Technol..

[30] Xu G., Diao P., He X., Wu J., Wang G., Wang C. (2019). Research on vehicle active steering control based on linear matrix inequality and hardware in the loop test scheme design and implement for active steering. Adv. Mech. Eng..

[31] Weir D.H. (2010). Application of a driving simulator to the development of in-vehicle human–machine-interfaces. IATSS Res..

[32] Galko C., Rossi R., Savatier X. Vehicle-Hardware-In-The-Loop system for ADAS prototyping and validation. Proceedings of the 2014 International Conference on Embedded Computer Systems: Architectures, Modeling, and Simulation (SAMOS XIV).

[33] Schiegg F.A., Krost J., Jesenski S., Frye J. A novel simulation framework for the design and testing of advanced driver assistance systems. Proceedings of the 2019 IEEE 90th Vehicular Technology Conference (VTC2019-Fall).

[34] Saraoğlu M., Morozov A., Janschek K. (2019). Safety assessment of autonomous and connected vehicles by a model-based traffic simulation framework. Proceedings of the 19. Internationales Stuttgarter Symposium: Automobil-und Motorentechnik.

[35] Lattarulo R., Pérez J., Dendaluce M. (2017). A complete framework for developing and testing automated driving controllers. IFAC-PapersOnLine.

[36] Sievers G., Seiger C., Peperhowe M., Krumm H., Graf S., Hanselmann H. Driving simulation technologies for sensor simulation in sil and hil environments. Proceedings of the DSC.

[37] Karl I., Berg G., Ruger F., Farber B. (2013). Driving behavior and simulator sickness while driving the vehicle in the loop: Validation of longitudinal driving behavior. IEEE Intell. Transp. Syst. Mag..

[38] Park C., Chung S., Lee H. (2020). Vehicle-in-the-loop in global coordinates for advanced driver assistance system. Appl. Sci..

[39] Shojaeefard M.H., Mollajafari M., Ebrahimi-Nejad S., Tayebi S. (2023). Weather-aware fuzzy adaptive cruise control: Dynamic reference signal design. Comput. Electr. Eng..

[40] Solmaz S., Rudigier M., Mischinger M. A vehicle-in-the-loop methodology for evaluating automated driving functions in virtual traffic. Proceedings of the 2020 IEEE Intelligent Vehicles Symposium (IV).

[41] Mihalič F., Truntič M., Hren A. (2022). Hardware-in-the-loop simulations: A historical overview of engineering challenges. Electronics.

[42] Soltani A., Assadian F. (2016). A hardware-in-the-loop facility for integrated vehicle dynamics control system design and validation. Ifac-Papersonline.

[43] Lee M., Lee H., Lee K.S., Ha S., Bae J., Park J., Park H., Choi H., Chun H. (2011). Development of a hardware in the loop simulation system for electric power steering in vehicles. Int. J. Automot. Technol..

[44] Eom H., Lee S.H. (2015). Human-automation interaction design for adaptive cruise control systems of ground vehicles. Sensors.

[45] Vo-Duy T., Ta M.C., Nguyễn B.H., Trovão J.P.F. (2020). Experimental platform for evaluation of on-board real-time motion controllers for electric vehicles. Energies.

[46] Chen Y., Chen S., Zhang T., Zhang S., Zheng N. Autonomous vehicle testing and validation platform: Integrated simulation system with hardware in the loop. Proceedings of the 2018 IEEE Intelligent Vehicles Symposium (IV).

[47] Tumasov A., Vashurin A., Trusov Y.P., Toropov E., Moshkov P., Kryaskov V., Vasilyev A. (2019). The application of hardware-in-the-loop (HIL) simulation for evaluation of active safety of vehicles equipped with electronic stability control (ESC) systems. Procedia Comput. Sci..

[48] Lee G., Ha S., Jung J.I. (2020). Integrating driving hardware-in-the-loop simulator with large-scale VANET simulator for evaluation of cooperative ECO-driving system. Electronics.

[49] Abboush M., Bamal D., Knieke C., Rausch A. (2022). Hardware-in-the-loop-based real-time fault injection framework for dynamic behavior analysis of automotive software systems. Sensors.

[50] Jan S.U., Lee Y.D., Shin J., Koo I. (2017). Sensor fault classification based on support vector machine and statistical time-domain features. IEEE Access.

[51] Amyan A., Abboush M., Knieke C., Rausch A. (2024). Automating Fault Test Cases Generation and Execution for Automotive Safety Validation via NLP and HIL Simulation. Sensors.

[52] Automotive Simulation Models (ASM). https://www.dspace.com/de/gmb/home/products/sw/automotive_simulation_models.cfm.

[53] dSPACE Software Tools. https://www.dspace.com/en/inc/home/products/products.cfm#.

[54] Abboush M., Knieke C., Rausch A. (2024). Representative Real-Time Dataset Generation Based on Automated Fault Injection and HIL Simulation for ML-Assisted Validation of Automotive Software Systems. Electronics.

[55] Zhou J., Schmied R., Sandalek A., Kokal H., del Re L. (2016). A framework for virtual testing of ADAS. SAE Int. J. Passeng. Cars-Electron. Electr. Syst..

[56] Avizienis A., Laprie J.C., Randell B., Landwehr C. (2004). Basic concepts and taxonomy of dependable and secure computing. IEEE Trans. Dependable Secur. Comput..

[57] Saeed U., Jan S.U., Lee Y.D., Koo I. (2021). Fault diagnosis based on extremely randomized trees in wireless sensor networks. Reliab. Eng. Syst. Saf..

